# Supplementary material for: Identifying disease genes using machine learning and gene functional similarities, assessed through Gene Ontology
Source: PLoS One. 2018 Dec 10;13(12):e0208626. doi: 10.1371/journal.pone.0208626 (PMC6287949; doi:10.1371/journal.pone.0208626)
Supplement: S1 Table — (DOCX) [file pone.0208626.s001.docx]

| **Semantic similarity measure** | **Classifiers** | **HD genes** | **HD + LD genes** | |
| --- | --- | --- | --- | --- |
|  |  | **Assessment 1** | **Assessment 1** | **Assessment 2** |
| **Resnik** | RF | 0.74 | 0.72 | 0.8 |
|  | NB | 0.67 | 0.65 | 0.75 |
|  | Linear-SVM | 0.28 | 0.62 | 0.66 |
|  | Radial-SVM | 0.52 | 0.62 | 0.6 |
| **Wang** | RF | 0.74 | 0.73 | 0.81 |
|  | NB | 0.68 | 0.65 | 0.75 |
|  | Linear-SVM | 0.27 | 0.63 | 0.65 |
|  | Radial-SVM | 0.54 | 0.61 | 0.59 |
| **Rel** | RF | 0.75 | 0.74 | 0.81 |
|  | NB | 0.68 | 0.65 | 0.76 |
|  | Linear-SVM | 0.29 | 0.64 | 0.65 |
|  | Radial-SVM | 0.45 | 0.62 | 0.6 |

S1 Table: The performance of classifiers over different semantic similarities matrices computed using Boot Mean Average (BMA) combining criteria.

HD: High confidence Disease genes; LD: Low confidence Disease genes
