# Supplementary material for: Identifying disease genes using machine learning and gene functional similarities, assessed through Gene Ontology
Source: PLoS One. 2018 Dec 10;13(12):e0208626. doi: 10.1371/journal.pone.0208626 (PMC6287949; doi:10.1371/journal.pone.0208626)
Supplement: S2 Table — (DOCX) [file pone.0208626.s002.docx]

| **Random Forest number of trees** | **Mean AUC value** |
| --- | --- |
| 200 | 0.78 |
| 300 | 0.78 |
| 400 | 0.78 |
| **500** | **0.8** |
| 600 | 0.79 |
| 700 | 0.78 |
| 800 | 0.78 |
| 1200 | 0.78 |
| 1800 | 0.79 |

S2 Table: The performance of Random Forest (RF) over different number of trees
